# Molecular diagnostic approach to rare neurological diseases from a clinician viewpoint

**DOI:** 10.1186/s44342-024-00025-0

**Published:** 2024-10-10

**Authors:** Jin Sook Lee

**Affiliations:** Department of Pediatrics, Seoul National University Hospital Child Cancer and Rare Disease Administration, Seoul National University Children’s Hospital, 101 Daehakro Jongno-gu, Seoul, 110-744 South Korea

**Keywords:** Rare disease, Neurological disease, Sequencing, Genomic test, Repeat expansion disorder

## Abstract

Advancements in sequencing technology have significantly enhanced diagnostic capabilities for rare neurological diseases. This progress in molecular diagnostics can greatly impact clinical management and facilitate the development of personalized treatments for patients with rare neurological diseases. Neurologists with expertise should raise clinical awareness, as phenotyping remains crucial for making a clinical diagnosis, even in the genomics era. They should prioritize different types of genomic tests, considering both the benefits and the limitations inherent to each test. Notably, long-read sequencing is being utilized in cases suspected to involve repeat expansion disorders or complex structural variants. Repeat expansion disorders are highly prevalent in neurological diseases, particularly within the ataxia group. Significant efforts, including periodic reanalysis, data sharing, or integration of genomics with multi-omics studies, should be directed toward cases that remain undiagnosed after standard next-generation sequencing.

## Introduction

Recent technological advances in sequencing have greatly improved diagnostic capabilities and facilitated the discovery of novel genes. This progress has deepened our understanding of pathomechanisms and ultimately led to the development of specific therapeutic options for rare diseases (RD). This is particularly true for rare neurogenetic diseases.

Approximately 80% of rare diseases have genetic origins, with nearly half of these conditions affecting the nervous system. About 80% of these rare diseases manifest in childhood, with more than 80% of the pediatric cases presenting with neurological symptoms. Given this context, neurologists with expertise should raise clinical awareness and be well-informed about available molecular diagnostic tests for rare neurogenetic diseases [1–5].

Even in the absence of definitive therapeutic options, establishing genetic diagnosis in rare neurological diseases can have significant implications for clinical management. It can provide valuable information about prognosis and complications. Besides that, it can pave the way for the discovery of novel biological pathways related to disease pathogenesis and the development of targeted therapies, ultimately contributing to the advancement of personalized medicine [6].

This mini-review aims to give an introduction to the molecular genetics of rare neurological diseases from a clinician’s perspective, with a particular focus on monogenic neurological diseases in children.

## Advances in molecular diagnostic approach to rare neurological diseases

### Prioritization of genomic tests

Rare neurogenetic diseases in childhood can be clinically classified into three groups: (1) neurodevelopmental disorders (e.g., Rett syndrome, Fragile X syndrome, or many other neurodevelopmental or neurodegenerative disorders), (2) movement disorders (e.g., hereditary spastic paraplegia or genetic ataxia), and (3) neuromuscular disorders (e.g., spinal muscular atrophy (SMA) or inherited peripheral neuropathy). Clinicians should prioritize various types of genomic tests for RD patients based on the clinical diagnosis, while also considering both the benefits and technical limitations inherent to each genomic test [2, 3].

For patients with global developmental delay, autism spectrum disorders, or multiple anomalies, the chromosomal microarray is used as a first-tier test in clinical practice. If the phenotype is very clear and the disease is caused by a single gene, single gene tests, such as Sanger sequencing or Multiplex ligation-dependent probe amplification (MLPA), are performed. For example, MLPA is performed for SMA. Sanger sequencing of the MECP2 gene is primarily used for Rett syndrome because MECP2 variants cause over 95% of typical Rett syndrome. However, most of the other neurological disorders have loci heterogeneity. More than 1000 genes are associated with neurodevelopmental disorders [6], over 100 genes with peripheral neuropathy [7], and more than 500 genes with epilepsy [8, 9]. Neurological diseases are known for phenotypic heterogeneity as well as genotypic heterogeneity. As many rare neurological diseases exhibit nonspecific symptoms, often with overlapping phenotypes, next-generation sequencing (NGS), such as targeted panel or whole exome sequencing (WES), is preferred. Whole genome sequencing (WGS) can provide uniform coverage and find structural variants or sequence alterations in non-coding regions [10], while it still has limitations regarding cost and interpretation of variants of uncertain significance (VUS).

### Utility of long-read sequencing in undiagnosed rare neurological diseases

More recently, long-read sequencing, i.e., PacBio Single-Molecule Real-Time sequencing or Oxford Nanopore Technology sequencing is being used for negative cases that are suspected to involve repeat expansion disorders or complex structural variants [11]. Repeat expansion disorders are estimated to have a prevalence of about 1 in 3000 people. Repeat expansion disorders are known to be about a total of 60 diseases to date [12], and are highly prevalent in neurological diseases (common repeat expansion disorders in childhood: see Table 1) [12–14]. Especially, genetic ataxia comprises most of the repeat expansion disorders. Targeted long-read sequencing or long-read whole genome sequencing (WGS) can find repeat expansion disorders missing from standard NGS or even short-read WGS. Long-read WGS for undiagnosed neurological diseases can identify repeat expansions in both coding and non-coding regions through a genome-wide approach [12]. Novel repeat expansion disorders have been discovered to be causative of ataxia [12, 15] and furthermore inherited neuropathy [7].

**Table 1:** Known neurological repeat expansion disorders in children

Diseases		Inheritance	Chromosome	Gene	Location, repeat motif	Pathomechanisms
Coding	SCA 1	AD	6p22	ATXN1	Exon, (CAG)n	Protein misfolding and aggregation
	SCA 2	AD	12q24	ATXN2	Exon, (CAG)n	Protein misfolding and aggregation
	SCA 3	AD	14q32	ATXN3	Exon, (CAG)n	Protein misfolding and aggregation
	SCA 6	AD	19p13	CACNA1A	Exon, (CAG)n	Protein misfolding and aggregation
	SCA 7	AD	3p14	ATXN7	Exon, (CAG)n	Protein misfolding and aggregation
	SCA 17	AD	6q27	TBP	Exon, (CAG)n	Protein misfolding and aggregation
	Huntington Disease	AD	4p16.3	HTT	Exon, (CAG)n	Protein misfolding and aggregation
	DRPLA	AD	12p13.31	ATN1	Exon, (CAG)n	Protein misfolding and aggregation
Non-coding	Fragile X syndrome	XLD	Xq27.3	FMR1	5′ UTR, (CGG)n	Gene silencing
	FRDA	AR	9q21.11	FXN	Intron, (GAA)n	Gene silencing
	myotonic dystrophy type 1	AD	19q13.32	DMPK	3′ UTR, (CTG)n	RNA toxicity, repeat-associated non-AUG translation
	SCA 8	AD	13q21	ATXN8OS/ATXN8	3′ UTR/exon, (CTG/CAG)n	RNA toxicity, repeat-associated non-AUG translation

*SCA* Spinocerebellar ataxia, *DRPLA* Dentatorubral-pallidoluysian atrophy, *FRDA* Friedreich ataxia

### Multidimensional strategies to overcome the diagnostic challenges

The diagnostic yield for rare neurological diseases is increasing with the help of advanced technology and various genomic tests. However, more than half of all suspected monogenic neurological diseases still remain undiagnosed. Significant efforts should be made in order to address the challenges [7, 16, 17]: (1) a periodic reanalysis, (2) data sharing, (3) advanced high-tech NGS, i.e., RNA sequencing in muscle diseases or single-cell sequencing, (4) multi-omics studies, and (5) functional studies using a mouse or organoid model. In addition to periodic reanalysis of genomic data, updating phenotypic information is particularly important in childhood, as phenotypic evolution can occur over time [18]. Data sharing with other genetics community can overcome the issue of rarity, the “n-of-1 problem” [16–20]. Various diagnostic modalities, such as RNA sequencing, single-cell sequencing, proteomics, or DNA methylomics, are being used in the research field. Integration of genomics with multi-omics studies can expand the knowledge of disease pathogenesis in known genes and facilitate the discovery of novel genes [16]. More recently, advanced cellular models for rare neurological diseases are being used, including induced pluripotent stem cells (iPSC) or 3D iPSC-derived organoids [21]. If no causative variant is reported, there might be non-genetic causes rather than genetic etiologies or the condition could be multifactorial or polygenic rather than monogenic. However, clinicians should approach negative cases with a perspective such as “beyond the exome” [10, 17]. Sometimes variant prioritization needs to be done “beyond documented inheritance modes”, as some genes are being identified to cause both dominant and recessive phenotypes [16, 22]. Multiple NGS strategies can be combined to overcome the limitations of each test.

### Importance of early diagnosis

Time to diagnosis is very crucial. Early diagnosis of probands with rare undiagnosed neurological diseases can lead their families to have genetic counseling and family planning. It is also critical for cases involving treatable neurometabolic disorders or rare neurological diseases with a narrow therapeutic window. Treatable NGS panels or rapid WES/WGS are being used for early identification of critically ill patients, such as those in the neonatal intensive care unit or pediatric intensive care unit.

### Therapeutic modalities in rare neurological diseases

Although the treatments are currently available in about 5–10% of total RD, there has been progress in therapeutic strategies, such as genetic therapy including gene transfer or gene editing [23]. Progress in molecular genetics of rare neurological diseases can lead to the establishment of personalized medicine, including newly approved treatments or emerging therapeutic options [13, 24]. Approved gene therapies include onasemnogene abeparvovec-xioi (Zolgensma) for the treatment of SMA in 2019 and eladocagene exuparvovec (Upstaza) for the treatment of aromatic L-amino acid decarboxylase deficiency in 2022 [25]. There are also two marketed intrathecally administered antisense oligonucleotides, nusinersen and tofersen, which were approved for the treatment of SMA and amyotrophic lateral sclerosis, respectively [26]. Other strategies, such as N of 1 clinical trial or drug repurposing, are being studied for rare neurological diseases [27].

## Conclusion

Advanced new technologies led to the improvement of diagnostic yield in rare neurological diseases. Even in the genomics era, phenotyping is key to both making a clinical diagnosis and properly selecting the genomic test. Long-read WGS can be used for unknown repeat expansion disorders or undiagnosed neurological diseases with complex genetic features, that are not detected by standard NGS techniques. Molecular diagnosis can facilitate clinical management and the development of personalized treatments for patients with rare neurological diseases.

## Data Availability

Not applicable.

## Data availability

No datasets were generated or analysed during the current study.

## References

[1] Di Resta C, Pipitone GB, Carrera P, Ferrari M. Current scenario of the genetic testing for rare neurological disorders exploiting next generation sequencing. Neural Regen Res. 2021;16(3):475–81. 32985468 10.4103/1673-5374.293135 PMC7996035

[2] Fellner A, Goldberg Y, Basel-Salmon L. Ordering genetic testing by neurologists: points to consider. J Neurol. 2023;270(8):3714–22. 37154893 10.1007/s00415-023-11758-3

[3] Jain V, Irving R, Williams A. Genomic testing in neurology. Pract Neurol. 2023;23(5):420–9. 37468300 10.1136/pn-2023-003735

[4] Papadopoulou E, Pepe G, Konitsiotis S, Chondrogiorgi M, Grigoriadis N, Kimiskidis VK, et al. The evolution of comprehensive genetic analysis in neurology: Implications for precision medicine. J Neurol Sci. 2023;447: 120609. 36905813 10.1016/j.jns.2023.120609

[5] Salunkhe M, Agarwal A, Faruq M, Srivastava AK. Genetic testing in neurology: what every neurologist must know. Ann Indian Acad Neurol. 2022;25(3):350–3. 35936600 10.4103/aian.aian_855_21 PMC9350807

[6] Parenti I, Rabaneda LG, Schoen H, Novarino G. Neurodevelopmental disorders: from genetics to functional pathways. Trends Neurosci. 2020;43(8):608–21. 32507511 10.1016/j.tins.2020.05.004

[7] Parmar JM, Laing NG, Kennerson ML, Ravenscroft G. Genetics of inherited peripheral neuropathies and the next frontier: looking backwards to progress forwards. J Neurol Neurosurg Psychiatry. Epub 2024 May 24. 10.1136/jnnp-2024-333436 PMC11503175 38744462

[8] Cavirani B, Spagnoli C, Caraffi SG, Cavalli A, Cesaroni CA, Cutillo G, et al. Genetic epilepsies and developmental epileptic encephalopathies with early onset: a multicenter study. Int J Mol Sci. 2024;25(2):1248. 38279250 10.3390/ijms25021248 PMC10816990

[9] Chang YT, Hong SY, Lin WD, Lin CH, Lin SS, Tsai FJ, et al. Genetic testing in children with developmental and epileptic encephalopathies: a review of advances in epilepsy genomics. Children (Basel). 2023;10(3). 10.3390/children10030556 PMC10047509 36980114

[10] Marwaha S, Knowles JW, Ashley EA. A guide for the diagnosis of rare and undiagnosed disease: beyond the exome. Genome Med. 2022;14(1):23. 35220969 10.1186/s13073-022-01026-w PMC8883622

[11] Oehler JB, Wright H, Stark Z, Mallett AJ, Schmitz U. The application of long-read sequencing in clinical settings. Hum Genomics. 2023;17(1):73. 37553611 10.1186/s40246-023-00522-3 PMC10410870

[12] Vegezzi E, Ishiura H, Bragg DC, Pellerin D, Magrinelli F, Currò R, et al. Neurological disorders caused by novel non-coding repeat expansions: clinical features and differential diagnosis. Lancet Neurol. 2024;23(7):725–39. 38876750 10.1016/S1474-4422(24)00167-4

[13] Depienne C, Mandel JL. 30 years of repeat expansion disorders: what have we learned and what are the remaining challenges? Am J Hum Genet. 2021;108(5):764–85. 33811808 10.1016/j.ajhg.2021.03.011 PMC8205997

[14] Malik I, Kelley CP, Wang ET, Todd PK. Molecular mechanisms underlying nucleotide repeat expansion disorders. Nat Rev Mol Cell Biol. 2021;22(9):589–607. 34140671 10.1038/s41580-021-00382-6 PMC9612635

[15] Kalia LV, Nimmo GAM, Mestre TA. Genetic testing in clinical movement disorders: a case-based review. Semin Neurol. 2023;43(1):147–55. 36854393 10.1055/s-0043-1763507

[16] Zech M, Winkelmann J. Next-generation sequencing and bioinformatics in rare movement disorders. Nat Rev Neurol. 2024;20(2):114–26. 38172289 10.1038/s41582-023-00909-9

[17] Schüle R, Timmann D, Erasmus CE, Reichbauer J, Wayand M, van de Warrenburg B, et al. Solving unsolved rare neurological diseases-a Solve-RD viewpoint. Eur J Hum Genet. 2021;29(9):1332–6. 33972714 10.1038/s41431-021-00901-1 PMC8440537

[18] Schobers G, Schieving JH, Yntema HG, Pennings M, Pfundt R, Derks R, et al. Reanalysis of exome negative patients with rare disease: a pragmatic workflow for diagnostic applications. Genome Med. 2022;14(1):66. 35710456 10.1186/s13073-022-01069-z PMC9204949

[19] Reinhard C, Bachoud-Lévi AC, Bäumer T, Bertini E, Brunelle A, Buizer AI, et al. The European reference network for rare neurological diseases. Front Neurol. 2020;11: 616569. 33519696 10.3389/fneur.2020.616569 PMC7840612

[20] Beijer D, Fogel BL, Beltran S, Danzi MC, Németh AH, Züchner S, et al. Standards of NGS data sharing and analysis in ataxias: recommendations by the NGS working group of the ataxia global initiative. Cerebellum. 2024;23(2):391–400. 36869969 10.1007/s12311-023-01537-1 PMC10951009

[21] Bombieri C, Corsi A, Trabetti E, Ruggiero A, Marchetto G, Vattemi G, et al. Advanced cellular models for rare disease study: exploring neural, muscle and skeletal organoids. Int J Mol Sci. 2024;25(2):1014. 38256087 10.3390/ijms25021014 PMC10815694

[22] Chung HL, Wangler MF, Marcogliese PC, Jo J, Ravenscroft TA, Zuo Z, et al. Loss- or gain-of-function mutations in ACOX1 cause axonal loss via different mechanisms. Neuron. 2020;106(4):589-606.e6. 32169171 10.1016/j.neuron.2020.02.021 PMC7289150

[23] FitzPatrick L, Bird A. Genetic therapies for neurological disorders. Hum Genet. 2022;141(5):1085–91. 34807307 10.1007/s00439-021-02399-5 PMC8607967

[24] Vogt L, Quiroz V, Ebrahimi-Fakhari D. Emerging therapies for childhood-onset movement disorders. Curr Opin Pediatr. 2024;36(3):331–41. 38655812 10.1097/MOP.0000000000001354 PMC11047116

[25] Ling Q, Herstine JA, Bradbury A, Gray SJ. AAV-based in vivo gene therapy for neurological disorders. Nat Rev Drug Discov. 2023;22(10):789–806. 37658167 10.1038/s41573-023-00766-7

[26] McCauley ME, Bennett CF. Antisense drugs for rare and ultra-rare genetic neurological diseases. Neuron. 2023;111(16):2465–8. 37354903 10.1016/j.neuron.2023.05.027

[27] Shah S, Dooms MM, Amaral-Garcia S, Igoillo-Esteve M. Current drug repurposing strategies for rare neurodegenerative disorders. Front Pharmacol. 2021;12: 768023. 34992533 10.3389/fphar.2021.768023 PMC8724568

